# Ameliorative effects of the melatonin on some cytokine levels, NF-κB immunoreactivity, and apoptosis in rats with cerulein-induced acute pancreatitis

**DOI:** 10.22038/IJBMS.2023.69019.15045

**Published:** 2024

**Authors:** Deniz Uluışık, Ercan Keskin, Tuğba Özaydın, Yasemin Öznurlu

**Affiliations:** 1 University of Selçuk, Faculty of Veterinary Medicine, Department of Physiology, Turkey; 2 University of Selçuk, Faculty of Veterinary Medicine, Department of Histology and Embryology, Turkey

**Keywords:** Acute pancreatitis, Apoptosis, Cytokine, Melatonin, NF-κB

## Abstract

**Objective(s):**

Investigating the ameliorative effects of melatonin on cytokine levels, apoptosis, and NF-κB immunoreactivity in rats with cerulein-induced acute pancreatitis.

**Materials and Methods:**

Thirthy-two Wistar Albino rats were divided into four groups: Control group which didn’t undergo acute pancreatitis induction and was left without treatment, pancreatitis group in which the acute pancreatitis was induced by 2 successive intraperitoneal doses of cerulein at a 2-hour interval (50 µg/kg and then 25 µg/kg), melatonin-treated pancreatitis group which was intraperitoneally administrated with 50 mg/kg of melatonin, 30 min before each cerulein injection, and melatonin group which was intraperitoneally administrated with 2 successive doses of melatonin (50 mg/kg each) at a 2-hour interval. Pancreatic tissue and blood samples were taken from animals of all groups. IL-1β, TNF-α, and IL-10 levels were determined in blood samples. Apoptosis was determined by the TUNEL assay and the NF-κB was detected immunohistochemically in acinar cells of the exocrine pancreatic portion.

**Results:**

IL-1β, TNF-α, and IL-10 levels in the acute pancreatitis group were significantly increased when compared to the control negative group. IL-1β and TNF-α levels in the melatonin-treated pancreatitis group were significantly lower than those of the acute pancreatitis group. While number of apoptotic cells and percentage of NF-κB immunopositive cells in the acute pancreatitis group were significantly increased compared to other groups and it was observed that these parameters were significantly reduced in the melatonin-treated pancreatitis group compared to the acute pancreatitis group.

**Conclusion:**

These findings suggest that melatonin administration can significantly reduce the severity of acute pancreatitis in rats.

## Introduction

Acute pancreatitis is a serious disease with an increasing incidence in humans. This disease causes varying degrees of organ dysfunction (1). The development and pathophysiology of pancreatitis in animals are similar to humans (2). Alcohol, hypercalcemia, hypertriglyceridemia, pancreatic duct obstruction, hereditary pancreatitis, dysfunction of the oddi sphincter, cystic fibrosis, autoimmune pancreatitis, ascariasis, ischemia, viral infections, embolism, and vasculitis are among the main causes of acute pancreatitis (1).

Most of the systemic disorders in acute pancreatitis are attributed to result in excessive systemic inflammatory responses by the entry of cytotoxic and inflammatory substances, cytokines, reactive oxygen species, and other mediators into the circulation by the release of these proteolytic enzymes (3, 4). Several studies have reported that ROS plays an important role in the early stages of this disease (5, 6).

Excessive production of ROS and decreased capacity of the intrinsic antioxidative defense system in acute pancreatitis cause peroxidation of lipid membranes, accumulation of ROS in the pancreas, and disruption of cell integrity (7). It has been reported that lipid peroxidation occurring in this process causes an increase in membrane permeability and ultimately cell death (8). It has been suggested that DNA fragmentation is provoked by proapoptotic gene bax and p53 expression and apoptosis is increased in relation to oxidative stress in acinar cells in acute pancreatitis (9, 10). In addition, the release of digestive enzymes into the pancreatic interstitium and thus their transition to the systemic circulation leads to an increase in cytokine production and release, playing an active role in making more destructive systemic and local effects (11).

It has been suggested that proinflammatory cytokines such as IL-1β, TNF-α, and IL-6 are local and systemic mediators and play a role in the occurrence of acute pancreatitis (12, 13). It has been reported that stimulated pancreatic macrophages increase the release of IL-1β and TNF-α in local tissue damage. IL-1β and TNF-α act locally and exacerbate acute pancreatitis. These cytokines together with IL-6 systemically increase capillary permeability, causing accumulation of leukocytes and extravasation that leads to multi-organ failure (10, 13).

Recent studies have shown that pancreatic acinar cell death occurs through both apoptosis and necrosis (14, 15). The severity of experimental pancreatitis correlated directly with the rate of necrosis and inversely with the rate of apoptosis. It is suggested that cerulein-induced acute pancreatitis in rats is characterized by low necrosis and relatively high apoptosis (15, 16).

It has been stated that the main source of ROS in acute inflammations is NADPH oxidases, and the main target of ROS and redox signal in acute pancreatitis is NF-κB (17, 18). It has been shown that both NF-κB and NF-κB-regulated IL-1β, IL-6, and TNF-α expressions are related to the onset and exacerbation of acute pancreatitis (19, 20). Therefore, it has been suggested that inhibition of ROS production in pancreatic acinar cells prevents adverse events caused by these cells by preventing inflammatory cell infiltration into pancreatic tissue (10).

Oxidative stress has been shown to be effective in the pathogenesis of pancreatitis and the stimulation of inflammatory signaling pathways (19-20). It is suggested that the use of antioxidants might be beneficial in acute pancreatitis in terms of reducing reactive oxygen species (ROS) levels (20). Melatonin, known as the hormone of darkness, is one of the substances that may be used for this purpose.

Melatonin was first discovered in the pineal gland in 1958 (5, 21, 22). Recent studies showed that melatonin is also present in large amounts in the gastrointestinal tract (23, 24). The plasma concentration of melatonin is at its maximum at night and its lowest during the day (22). Melatonin is well tolerated without toxic effects and is a potent free radical scavenger and tissue damage inhibitor (25). Melatonin easily penetrates the cell due to being a highly lipophilic substance (22). Also, melatonin has the ability to activate the immune defense system. It has been shown that melatonin increases the proliferation of immune cells, and the life span of granulocytes and B lymphocytes and modulates cytokine production with nitric oxide and prostaglandin formation (22, 26). 

It has been suggested that melatonin modulates the production of cytokines through inhibition of the NF-κB transcription factor, which is related to immunity, inflammation, prostaglandin production, cell adhesion molecules, cytokines, and apoptosis inhibitors (5, 7). In relation to NF-κB, it has been reported that melatonin regulates apoptosis and necrosis processes, stimulates vascular endothelial growth factor (VEGF) production, and accelerates the angiogenesis process and all these properties mediate the positive effects in acute pancreatitis (7, 24). However, it is emphasized that there may be various ways to limit the progression of this disease through the immunoregulatory properties of melatonin (5, 26).

High doses of cerulein, a cholecystokinin (CCK) analog, have been reported to stimulate the maximum secretion of pancreatic amylase and lipase (27). The increase in secretion results in pancreatitis, which is characterized by cytoplasmic vacuolization, the death of acinar cells, edema formation, and infiltration of inflammatory cells into the pancreas (20, 28).

This study was designed to determine whether the melatonin pretreatment has ameliorative effects on some cytokine levels and NF-κB immunoreactivity in rats with cerulein-induced acute pancreatitis.

## Materials and Methods

***Experimental design***

Thirty-two healthy adult male Wistar albino rats were used in the study. During the experimental period, the optimal conditions for the rats were provided. All animals were allowed to drink water while fasting for 16 HR before the start of the study. The rats were divided into four groups as follows: control group (C, n=6) the rats that did not undergo acute pancreatitis induction and were left without treatment, pancreatitis group (P, n=10) the rats that underwent acute pancreatitis induction by 2 successive intraperitoneal doses of cerulein at a 2-hour interval (50 µg/kg and then 25 µg/kg); melatonin-treated pancreatitis group (MP, n=10) in which the rats were intraperitoneally administrated with 50 mg/kg of melatonin 30 min before each cerulein injection, and melatonin group (M, n=6) in which the rats were administrated with 2 successive intraperitoneal injections of melatonin (50 mg/kg each) at a 2-hour intervals.

***Blood sampling and detection of IL-1β, TNF-α and IL-10 levels***

Blood samples were taken from animals of all groups 12 hr after the last cerulein injection. The levels of IL-1β, TNF-α, and IL-10 were determined by an ELISA test (Biotek ELx800, Biotek Instrumentations, Inc, Winooski, VT, USA) using a commercial kit (Elabscience).

***Tissue specimen collection and staining***

After animal sacrification via cervical dislocation, pancreatic tissue specimens were taken and ﬁxed in 10% formalin for 24 hr, underwent a series of histological preparations, and were sectioned using a rotary microtome into 6 μm thick tissue sections according to literature (29, 30). The sections were stained with Crossmon’s triple stain according to Crossmon (31).

***Detection of apoptosis in the pancreatic cells by the TUNEL Assay***

Apoptotic cells were detected by enzymatic labeling of DNA strand breaks by using the TUNEL method according to literature (32, 33)(Calbiochem QIA33 was used). The DNA fragmentation detection kit used in this study contains HL-60 control slides. HL60 control slides that contain a mixture of HL60 cells incubated with 0.5 µg/ml actinomycin D for 19 hr to induce apoptosis and HL60 cells uninduced were stained as positive control slides according to the protocols of the manufacturer.

***Immunohistochemical detection of the NF-*** ***κB*** ***in acinar cells of the pancreas***

NF-κB p65 was stained immunohistochemically using a sensitive peroxidase-labelled streptavidin-biotin detection system. The sections were dewaxed in a xylene series and rehydrated. In order to unmask the antigen, the sections were placed into 10 mM citric acid buffer (pH 6.0) and heated in a microwave oven (700 W for 5 min). Endogenous peroxidase activity was blocked with 3% hydrogen peroxide in methanol for 20 min. Nonspecific binding sites were blocked by incubating the sections in a blocking solution (ScyTek UHP 125, USA) for 20 min. The sections were then incubated with the following primary antibodies: Anti-NF-κB p65 antibody (ab16502; 1:1000 dilution) for 1 HR at room temperature (RT). They were then treated with a biotinylated goat antimouse secondary antibody (IgG) (ScyTek UHP 125, USA) for 20 min at RT, followed by treatment with horseradish peroxidase (HRP)-streptavidin (ScyTek UHP 125, USA) for 20 min at RT. The color reaction was developed with 3,3ʹ-diaminobenzidine (DAB) (ScyTek UHP 125, USA). The slides were counterstained with Mayer’s hematoxylin for 1 min and then mounted with synthetic resin. In the negative control slides, the tissue sections were incubated with PBS without the primary antibodies.

The specimens of all groups were examined under a light microscope (Leica DM2500) and then were photographed by a digital camera (Leica DFC 320). In the sections stained with the TUNEL method, five fields were photographed with x200 magnification, and all apoptotic cells at these fields were counted. The sections that were stained with the NF-κB p65 immunostaining method were photographed at x400 magnification. The NF-κB p65 positive aciner cells were counted in randomly selected ten different areas of 10000 µm2 in each field and the percentages of NF-κB p65 positive cells were calculated from the ratio between the number of positively immunostained cells and the total number of counted cells x100.

***Statistical Analysis***

The data were analyzed by one-way ANOVA and Duncan’s multiple range test (SPSS 19.0, SPSS Inc., Chicago, Ill, USA).

## Results

The effects of melatonin administration on cytokine levels, apoptotic cell numbers, and NF-κB p65 immunopositive cell percentages in rats with cerulein-induced acute pancreatitis are given in Table 1.

***Plasma cytokine levels***

TNF-α, IL-1β, and IL-10 levels increased significantly (*P*<0.05, Table 1) in pancreatitis compared to the control group. TNF-α and IL-1β levels in the melatonin-treated pancreatitis group were significantly lower than in the pancreatitis group (*P*<0.05, Table 1). The change in IL-10 level with melatonin administration was not statistically significant.

***Histological findings***

**Figure 1:**
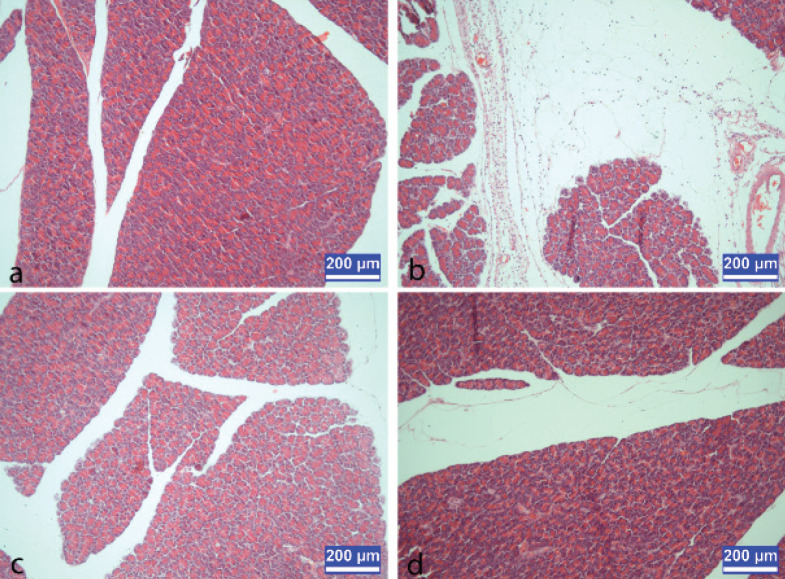
a) control group, b) pancreatitis group rat c) melatonin-treated pancreatitis group rat d) melatonin group rat

The histological examination of the pancreas showed a normal histological structure in the control and melatonin groups (Figure 1a, 1d). Intense edema and inflammation were seen in the pancreatitis group (Figure 1b), whereas the edema and inflammation formation were improved in the melatonin treatment group (Figure 1c).

***Apoptosis in the pancreatic cells***

**Figure 2:**
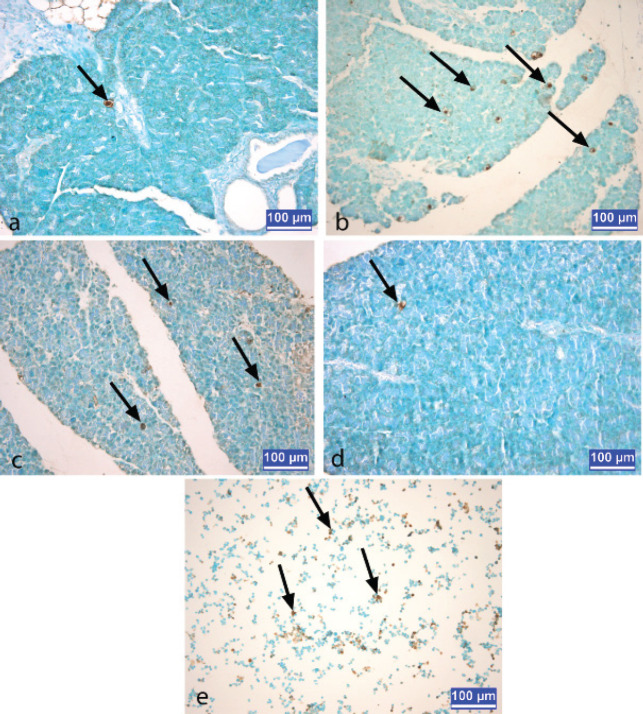
a) control group rat b) pancreatitis group rat c) melatonin-treated pancreatitis group rat d) melatonin group rat e) HL60cells as positive control slide

In the sections stained by the TUNEL method, the cells with brown-stained nuclei were classified as apoptotic cells*. *While the mean apoptotic cell number was highest in the pancreatitis group, this value was significantly decreased (*P*<0.05) in the melatonin-treated pancreatitis group (Figure 2, Table 1).

***Immunohistochemical findings***

**Figure 3:**
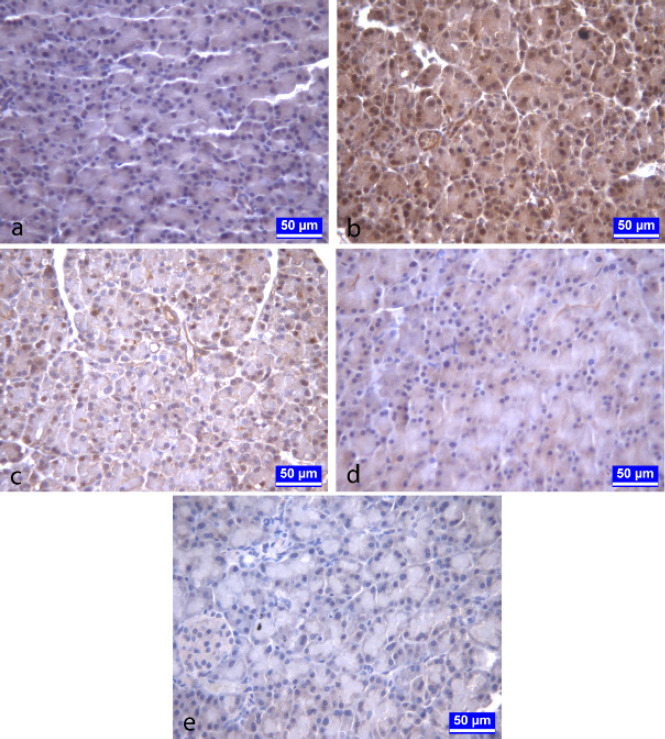
a) control group rat b) pancreatitis group rat c) melatonin-treated pancreatitis group rat d) melatonin group rat e) Negative control slide

The NF-κB p65 immunopositivity was observed in nuclei of pancreatic acinar cells. While the NF-κB p65 immunopositive cell percentage was highest in the acute pancreatitis group, this percentage was significantly decreased (*P*<0.05) in the melatonin-treated pancreatitis group in comparison with the pancreatitis group (Figure 3, Table 1).

**Table 1:** Cytokine levels, apoptotic cell numbers, and NF-κB p65 immunopositive cell percentages (Mean±SE)

**Groups**	**IL-1β** **(pg/ml)**	**TNF-α** **(pg/ml)**	**IL-10** **(pg/ml)**	**Apoptotic cell numbers**	**NF-κB p65 immunopositive cell percentages**
**C**	51,28±2,12^c^	82,58±1,52^c^	54,68±1,07^b^	0.53±0.15^c^	0,21±0.10^c^
**P**	93,78±5,16^a^	141,36±3,08^a^	65,77±1,23^a^	12.25±0.92^a^	53.52±1.78^a^
**MP**	74,93±2,50^b^	107,07±3,70^b^	69,26±1,85^a^	7.42±0.92^b^	14,62±1,45^b^
**M**	**47,43±2,17** ^c^	**84,87±1,77** ^c^	**58,79±0,91** ^b^	**0.50±0.15** ^c^	**0,18±0,11** ^c^

^a-c^ Within the same column, values with different small alphabetical superscripts are significant variables (*P<*0.05)

C: control, P: pancreatitis, MP: melatonin-treated pancreatitis groups

M: melatonin

Normal histological structures were observed in the control and melatonin groups (a, d) Intense edema and inflammation were seen in the pancreatitis group (b), whereas edema and inflammation formation were improved in the melatonin treatment group (c) Crosmans’s triple stain

Arrows: Apoptotic cells. Many apoptotic cells were observed in the pancreatitis group (b) Apoptotic cells in the melatonin-treated pancreatitis group (c) were less than in the pancreatitis group. TUNEL staining

No immunohistochemical staining was observed in the negative control slide. Many NF-κB p65 immunopositive cells were observed in the acute pancreatitis group (b) NF-κB p65 immunopositive cells in the melatonin-treated pancreatitis group (c) were less than in the pancreatitis group. NF-κB p65 immunohistochemical staining

## Discussion

Acute pancreatitis is an edematous disease and has a lethal prognosis. This disease is characterized by leukocyte activation, diffuse inflammatory cell infiltration, activation of digestive proteases, release of various inflammatory mediators, and acinar cell necrosis (3). Data from Sternby *et al.*’s (34) study demonstrate a distinct change in IL-1β, IL-8, IL-10, and IL-6 over the first 48 hr after onset of acute pancreatitis. Similarly, a study (35) suggested that the levels of TNF-α, IL-1β, and IL-6 were increased in acute pancreatitis groups compared with the sham group. In addition, the researchers reported that the expression of IL-10 was slightly increased compared with the sham group. It was reported that proinflammatory cytokines such as TNF-α and IL-6 levels significantly increased in acute pancreatitis (10, 12, 13). Also, a study (13) stated that TNF-α production was significantly increased in pancreatitis rats as compared with non pancreatitis control rats. The TNF-α, IL-1β, and IL-10 increased significantly in rats of the pancreatitis group of this study compared to healthy rats, they are considered indicators for acute pancreatitis and this finding is consistent with the findings of other authors (10, 12, 13, 34, 35).

Studies have reported that melatonin increases the amount of various antioxidant enzymes and limits inflammatory mediators in experimental pancreatitis (36-38). In relation to this, it is reported that melatonin increases nuclear factor erythroid 2-related factor expression and decreases the expressions of IL-1β, IL-6, IL-8, TNF-α, and iNOS (7). Ali and Madkour (36) suggested that the tissue concentrations of TNF-α, IL-6, and IL-1β in the melatonin pretreatment group were significantly lower than in the L-arginine-induced acute pancreatitis group. In the group of acute pancreatitis, treatment with melatonin reduced the increases in IL-6 and TNF-α levels caused by acute pancreatitis according to the results of Le *et al.* (38). In the present study, TNF-α and IL-1β levels were significantly lower than those of the pancreatitis group as a result of pre-application of melatonin. These results are in accordance with the earlier reports which demonstrated that melatonin pretreatment in pancreatitis improved the proinflammatory cytokines (7, 24, 39, 40). Also, it has been stated that proinflammatory cytokines (TNFα, IL-1β, and IL-6) were significantly reduced, whereas antiinflammatory cytokines (IL-10 and IL-4) were increased in animals with acute pancreatitis pretreated with melatonin (5, 8, 41-45). Ali and Madkour (36) also stated that the IL-10 level in the melatonin pretreatment of rats with acute pancreatitis was significantly increased. In our study, IL-10 levels increased with melatonin administration, but this increase was not statistically significant. In various studies, it has been suggested that melatonin is useful in the prevention, alleviation, or treatment of some diseases. Due to the antioxidant, anti-inflammatory, and immunoregulatory properties of melatonin, it is thought that its use may be beneficial for the prevention of complications such as multisystem organ failure, sepsis, and necrosis that may be caused by acute pancreatitis (5, 46-48).

NF-κB modulates the expression of many genes that play a key role in apoptosis, inflammation, viral replication, and tumorigenesis. NF-κB is inactive in the cytoplasm of unstimulated cells and when activated, it translocates to the nucleus where it can activate the transcription of its target genes (49). A study reported that NF-kB is rapidly activated in acute pancreatitis (50). Besides, Huang *et al.* (51) suggested that NF-κB activation is associated with the severity of acute pancreatitis. Another study (52) stated that cerulein exposures caused increased pancreatic apoptosis and NF-κB p65 expression. Similarly, higher levels of NF-κB p65 expression and increased pancreatic apoptosis have been reported in pancreatic tissue of the severe acute pancreatitis group of rats (53). Other studies have also reported that experimentally induced acute pancreatitis causes apoptosis and increased NF-κB activation in acinar cells of the pancreas (51, 54). In this study, the NF-κB p65 immunopositivity was observed in nuclei of pancreatic acinar cells and it was determined that NF-κB p65 immunopositive cell percentage and apoptotic cell number were highest in the pancreatitis group.

Previous studies have shown that pretreatment with melatonin in rats with acute pancreatitis could prevent pancreatic inflammation and radically reduce pancreatic tissue damage (5, 7, 8, 42-45, 55, 56). In histological assessment of pancreatic tissue, melatonin caused prominent decline of the inflammatory markers such as edema and neutrophil infiltration (8, 55, 57, 58). Consistent with these studies, melatonin administration to rats with cerulein-induced pancreatitis improved the edema and inflammation formation in this study.

It has been suggested that melatonin administration to acute pancreatitis caused by ischemia/reperfusion (IR) injury prevented all tissue markers of oxidative stress, biochemical and histological signs of apoptosis and necrosis, and restored glandular function (7, 26, 43, 59). In the present study, melatonin administration to rats with cerulein-induced pancreatitis significantly decreased the NF-***κB*** p65 immunopositive cell percentages and apoptotic cell numbers in pancreatic tissue. The above studies and our findings revealed that pretreatment with melatonin significantly decreases inflammation in pancreatic tissue as well as reducing apoptosis in pancreatitis. It is known that NF-κB plays an important role in the development of acute pancreatitis by regulating the synthesis of some cytokines. The reduction in the severity of acute pancreatitis with melatonin treatment may be explained by decreased NF-κB activation.

In addition to the plasma cytokine levels and histological findings determined in this study, further studies are needed to determine different parameters in blood and pancreatic tissue to evaluate the effects of melatonin pre-application in the prevention and reduction of acute pancreatitis.

## Conclusion

As a result, it was concluded that melatonin pre-application in rats with cerulein-induced acute pancreatitis might reduce the severity of acute pancreatitis by decreasing NF-κB p65 expression and apoptotic cell number in pancreatic tissue and plasma proinflamatuar cytokine levels. In addition to the plasma cytokine levels and histological findings determined in this study, further studies are needed to determine different parameters in blood and pancreatic tissue to evaluate the effects of melatonin pre-application in the prevention and reduction of acute pancreatitis.

## Authors’ Contributions

D U and E K contributed to the conception, design, acquisition, analysis, and interpretation, drafted the manuscript, and gave the final approval. T Ö and Y Ö contributed to the analysis and interpretation and critically revised the manuscript. All authors have read and approved the final manuscript.

## Ethical Approval

The study was approved by the Ethics Committee of Selçuk University Experimental Medicine Research and Application Center, Türkiye (Report No. 2017-16).

## Conflicts of Interest

The authors have no conflicts of interest to declare.
